# A comprehensive understanding of the chemical vapour deposition of cadmium chalcogenides using Cd[(C_6_H_5_)_2_PSSe]_2_ single-source precursor: a density functional theory approach

**DOI:** 10.1186/s13065-016-0146-3

**Published:** 2016-02-02

**Authors:** Francis Opoku, Noah Kyame Asare-Donkor, Anthony Adimado Adimado

**Affiliations:** Department of Chemistry, Kwame Nkrumah University of Science and Technology, Kumasi, Ghana

**Keywords:** Chemical vapour deposition, Chalcogenides, Phosphinato, Decomposition, Potential energy surface

## Abstract

**Background:**

The phosphinato complexes of group IIB are of great interest for their potential toward technological applications. A gas phase mechanistic investigation of the chemical vapour deposition of cadmium chalcogenides from the decomposition of Cd[(C_6_H_5_)_2_PSSe]_2_, as a single source precursor is carried out and reported herein within the framework of density functional theory at the M06/LACVP* level of theory.

**Results:**

The results reveal that the activation barriers and the product stabilities on the singlet potential energy surface (PES) favour CdS decomposition pathways, respectively. However, on the doublet PES, the activation barriers favour CdS while the product stabilities favour CdSe decomposition pathways, respectively. Contrary to the previously reported theoretical result for Cd[(^*i*^Pr)_2_PSSe]_2_, CdSe decomposition pathways were found to be the major pathways on both the singlet and the doublet PESs, respectively.

**Conclusion:**

Exploration of the complex gas phase mechanism and a detailed identification of the reaction intermediates enable us to understand and optimise selective growth process that occur in a chemical vapour deposition.

**Graphical abstract:**
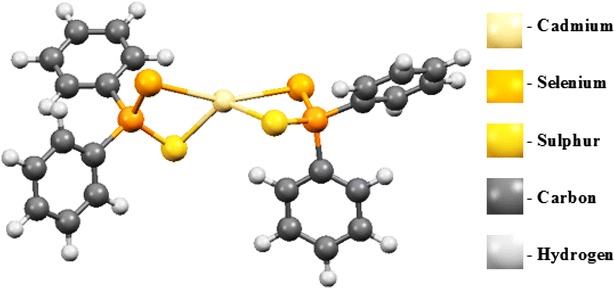
Structure of Cd[(C_6_H_5_)_2_PSSe]_2_ single-source precursor

## Background

The chemical and coordinating properties of anionic ligands R_2_PCh_2_^−^ with phosphorus, sulphur and selenium donor atoms (Ch = S, Se) are well documented [1–6]. Dithiophosphinates R_2_PS_2_^−^ and diselenophosphinates R_2_PSe_2_^−^, where R = alkyl or aryl, are known and widely used as single source precursors of remarkable nanomaterials [7–10] and ligands for metal complexes [11–18]. Moreover, thioselenophosphinates represent rare anionic conjugate triads of “S-P-Se” type, possessing of *S,Se*-ambident reactivity, a type of compounds which is nearly unexplored [19–25].

II–VI nanostructure semiconductors have been of considerable interest in the past decade due to their unique optical and electrical properties, and good candidates for the building blocks of functional Nano devices such as field-effect transistors (FETs), [26, 27] photo detectors (PDs), [28, 29] light-emitting diodes (LEDs), [30] photovoltaic (PV) devices [31, 32] and logic circuits [33, 34]. Semiconductor materials such as CdSe, CdTe, and CdSe_x_Te_1−x_ are the bases of modern electronic devices. CdSe is one of the most promising semiconducting materials with potential applications in solar cells, [35, 36] γ-ray detectors, [37] thin film transistors, [38] etc. Doped semiconductor Nano crystals with transition metals have attracted much attention due to their unique properties [39–41].

Gas-phase chemistry for the chemical vapour deposition (CVD) of metal precursors has been the subjects of theoretical investigations as gas-phase reactions, in particular, are found to play a key role in CVD process which has a number of important industrial and commercial applications. Theoretical data on single-source precursor bearing the thioselenophosphinate groups, [R_2_PSeS], are lacking in literature. Very recently, we have undertaken a theoretical study on several single source precursors (SSPs) to deposit metal chalcogenides via the gas phase decomposition process [42–46]. Spurred by the success of the use of SSPs and motivated by their potential to reduce the environmental impact of material processing, we have been keenly interested in investigating new routes to prepare SSPs. In addition, ligands binding strength on single-source metal precursor can be employed to tune the decomposition kinetics of the complex. Contrary, multiple-source routes often use highly toxic and/or oxygen or moisture sensitive gases, or very volatile ligands, such as: (CH_3_)_2_Cd (Et_3_)_3_Ga, H_2_E (E = S or Se) or EH_3_ (E = N, P or As).

In continuation of our research into thioselenophosphinato metal complexes, we have investigated the possibility of the gas phase decomposition of single source precursors within Cd[(C_6_H_5_)_2_PSSe]_2_ complex. To gain insight into the complete reaction features, theoretically we have employed density functional theory technique. The reaction kinetics is also studied, employing standard transition state theory to evaluate the rate constant of the elementary reactions involved.

### Computational details

All calculations were carried out with Spartan‘10 v1.1.0 Molecular Modelling program [47] at the DFT M06/LACVP* level in order to maximize the accuracy on the chemically active electrons of the reactions while minimizing computational time. LACVP* basis set uses the Hay–Wadt ECP basis set for cadmium, [48] and the 6-31G* basis set for all other atoms [49] as implemented in Spartan [47]. Zhao and Truhlar [50] recently developed the M06 family of local (M06-L) and hybrid (M06, M06-2X) meta-GGA functionals that show promising performance for the kinetic and thermodynamic calculations without the need to refine the energies by post Hartree–Fock methods. The M06 is reported to show excellent performance for transition metal energetics [50] and is therefore strongly recommended for transition metal chemistry [51].

The starting geometries of the molecular systems were constructed using Spartan’s graphical model builder and minimized interactively using the sybyl force field [52]. The equilibrium geometries of all molecular species were fully optimized without any symmetry constraints. Frequency calculations were carried out for all the stationary points at the corresponding level of theory to characterize the optimized structures as local minima (no imaginary frequency) or as transition states (one imaginary frequency) on the potential energy surfaces. The connecting first-order saddle points, the transition states between the equilibrium geometries, are obtained using a series of constrained geometry optimization in which the breaking bonds were fixed at various lengths and optimized the remaining internal coordinates.

The rate constants were computed using the transition state theory for the selected reaction pathways [53, 54].

1 $$k_{uni} = \left( \frac{{\kappa k_{B} T}}{h} \right)\exp^{ - \left( \frac{\Updelta G^{^\ddag} }{RT} \right)}$$

where ΔG^‡^ is the activation free-energy, ΔG^o^ is the Gibbs free energy, and k_B_ and h are the Boltzmann and Planck constants, respectively.

### Mechanistic considerations

**Scheme 1:**
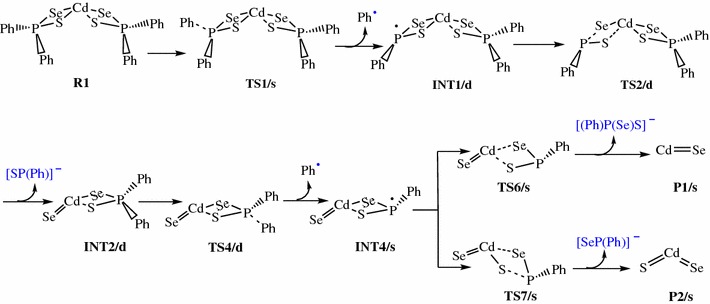
Proposed decomposition pathway of (C_6_H_5_)P(Se)S-Cd-Se intermediate [38–41]

**Scheme 2:**
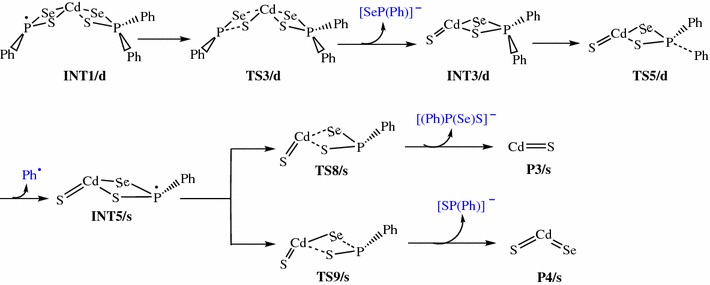
Proposed decomposition pathway of (C_6_H_5_)P(Se)S-Cd-S intermediate [38–41]

**Scheme 3:**
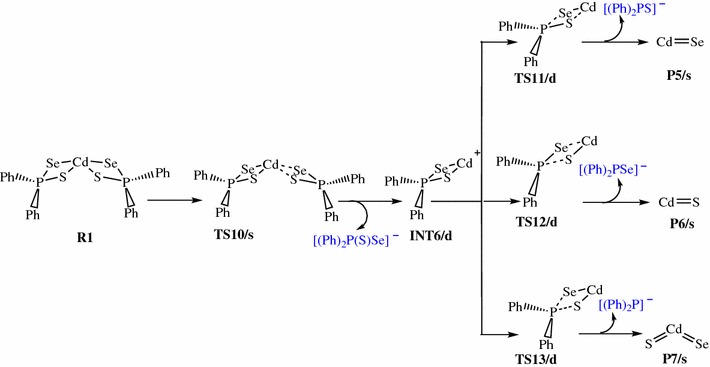
Proposed decomposition pathway of (C_6_H_5_)_2_P(Se)S-Cd intermediate [38–41]

**Scheme 4:**
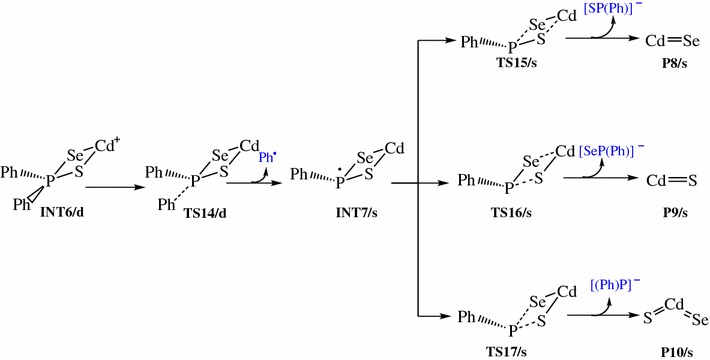
Proposed decomposition pathway of (C_6_H_5_)P(Se)S-Cd intermediate [38–41]

The reaction pathways for the gas phase decomposition of Cd[(C_6_H_5_)_2_PSSe]_2_ complex were based on the possible routes suggested Akhtar et al. [55] and Opoku et al. [42–46]. Schemes 1, 2, 3, 4 takes into account all these probable theoretically investigated decomposition pathways.

## Results and discussion

### Optimized geometry of Cd[(C_6_H_5_)_2_PSSe]_2_ precursor

Table 1 shows the M06/LACVP* calculated geometries for the Cd[(C_6_H_5_)_2_PSSe]_2_ and Cd[(^*i*^Pr)_2_PSSe]_2_ precursors. The Cd–Se bond lengths are in the range of 2.99–3.02 Å which are slightly longer than the Cd[(^*i*^Pr)_2_PSSe]_2_ precursor 2.81 Å [42]. The bond angle of Se_1_–Cd–S_1_ (79.1°) is more acute than the Se–Cd–Se angle in Cd[(SeP^*i*^Pr_2_)_2_N]_2_ [111.32(6)u] [56]. The average Cd–Se bond lengths, 3.01 Å, as expected are longer than the Cd–S distance, 2.59 Å. The S–Cd–Se angle (79°) is smaller than the S–P–Se angle (119°) due to the large amount of repulsion between the lone pairs of electrons of phosphorus with those of cadmium. The wider Se_1_–Cd–Se_2_ bond angle of 159.4° was as a result of the proximity of the non-coordinating Se-donor atoms to the Cd(II) atom.

**Table 1:** Comparison of the calculated geometries of Cd[(C_6_H_5_)_2_PSSe]_2_ and Cd[(^*i*^Pr)_2_PSSe]_2_ precursor at the M06/LACVP* level of theory (bond lengths in angstroms and bond angles in degrees)

Bond lengths	M06/LACVP*		Bond angles	M06/LACVP*	
P_1_–Se_1_	2.10	2.20^a^	Se_1_–P_1_–S_1_	118.5	112.3^a^
P_1_–S_1_	2.05	2.07^a^	S_2_–P_2_–Se_2_	118.7	112.2
S_2_–P_2_	2.01	2.07^a^	Se_1_–Cd–S_1_	79.1	83.5^a^
Se_2_–P_2_	2.11	2.20^a^	S_2_–Cd–Se_2_	79.1	83.3^a^
Cd–Se_1_	3.02	2.81^a^	Se_1_–Cd–Se_2_	159.4	124.9^a^
Cd–S_1_	2.57	2.51^a^	S_1_–Cd–S_2_	124.0	119.6^a^
Se_2_–Cd	2.99	2.81^a^	Se_2_–Cd–S_2_	104.4	116.4^a^
S_2_–Cd	2.61	2.51^a^	S_1_–Cd–Se_2_	116.4	133.0^a^

^a^Data from Ref. [38]

The geometry around P_1_ and P_2_ is a distorted tetrahedral (Se_1_–P_1_–S_1_ and S_2_–P_2_–Se_2_: 118.5 and 118.7). The structure of Cd[(C_6_H_5_)_2_PSSe]_2_ precursor adopts a symmetric and puckered macro cyclic framework, with the two phenyl rings directly attached to phosphorus atoms being parallel to each other. The Se–P–Se bond angles are enlarged from ideal tetrahedral Se_1_–P_1_–S_1_ and S_2_–P_2_–Se_2_: 118.5 and 118.7, respectively, and are considerably slightly larger than those in Cd[(^*i*^Pr)_2_PSSe]_2_ precursor [112.3 and 112.3] [42].

### Overall decomposition of Cd[(C_6_H_5_)_2_PSSe]_2_ precursor

**Fig. 1:**
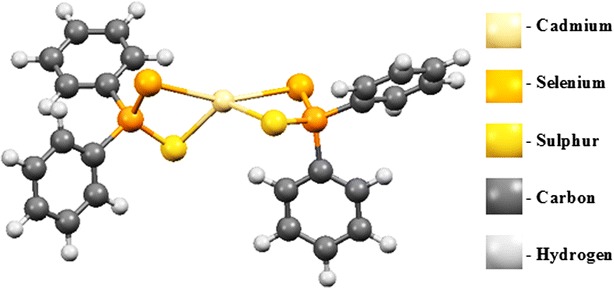
Structure of Cd[(C_6_H_5_)_2_PSSe]_2_ single-source precursor

**Fig. 2:**
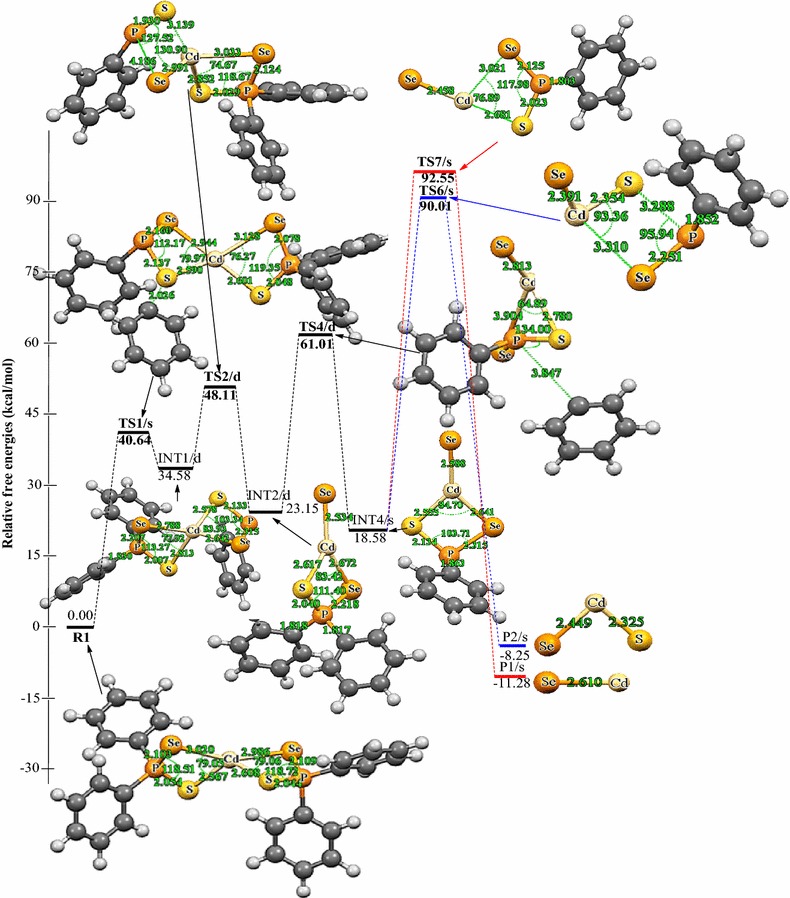
Energy profile of the decomposition pathway of (C_6_H_5_)PSSe–Cd–Se intermediate. Data in the path are the relative Gibbs free energies (in kcal/mol and bond distances in angstroms) obtained at M06/6-31G(d) level

The following discussions are aimed at elucidating the detailed mechanistic scenario and thereby providing a molecular level understanding of the complete reaction features associated with Cd[(C_6_H_5_)_2_PSSe]_2_ precursor. Twenty four reactions have been investigated in total: seven energy minima and seventeen transition states. The relative energies and the optimized geometries of all the species involved in the (C_6_H_5_)PSSe–Cd–Se and (C_6_H_5_)PSSe–Cd–S decomposition are depicted in Figs. 1 and 2.

Unimolecular decomposition of R1 via pathway 1 is associated with the elimination of phenyl radical leading to the formation of a (C_6_H_5_)_2_PSSe–Cd–SeSP(C_6_H_5_) intermediate, INT1/d (Fig. 2). This dissociation pathway passes through a singlet transition state TS1/s with a barrier height of 40.64 kcal/mol and reaction energy of 34.58 above the initial reactant on the doublet potential energy surface. This barrier is significantly lower than the barrier for the formation of the (^*i*^Pr)_2_PSSe–Cd–SeSP(^*i*^Pr) intermediate (∼77 kcal/mol) [42].

A doublet transition state was obtained for the (C_6_H_5_)_2_PSSe–Cd–Se intermediate, INT2/d and was found to be 3.93 kcal/mol lower than the (C_6_H_5_)_2_PSSe–Cd–S intermediate INT3/d. This process is found to be exergonic, producing INT2/d at an energy level of 11.43 kcal/mol below the initial intermediate, INT1/d. A doublet transition state, TS2/d, located for this conversion, is a four-membered cyclic transition state and involves the dissociation of the Cd–S and P–Se bonds. In TS2/d, the Cd–S and P–Se bonds are elongated by 0.35 and 2.18 Å, respectively relative to the initial intermediate, INT1/d. The formation of the (C_6_H_5_)_2_PSSe-Cd-S intermediate, INT3/d via a doublet transition state TS3/d has an activation barrier and a relative free energy of 17.46 and 4.50 kcal/mol, respectively below INT1/d.

**Fig. 3:**
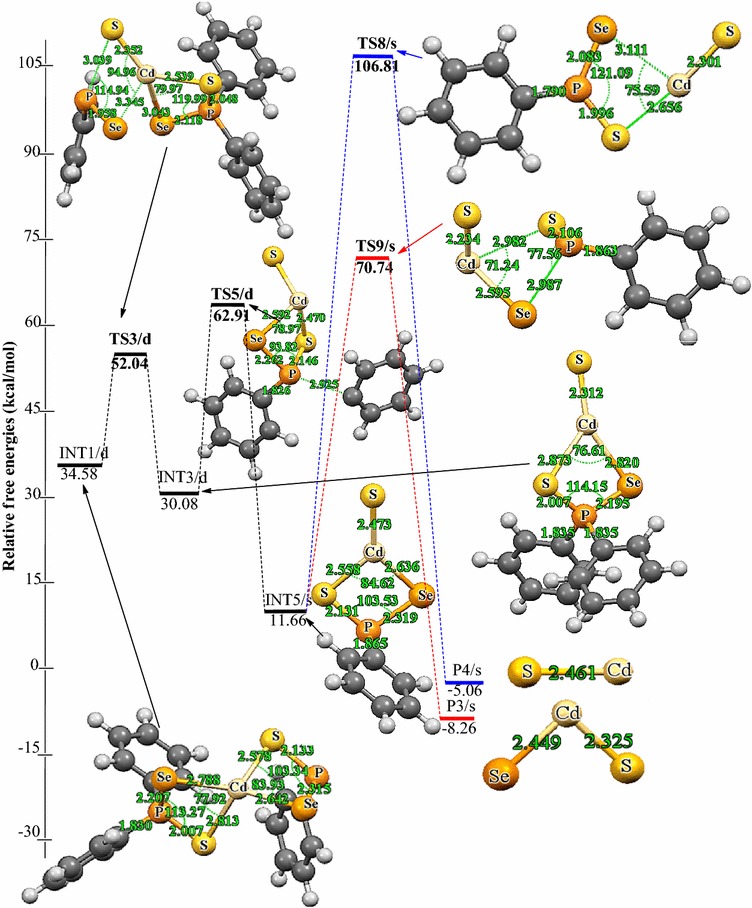
Energy profile of the decomposition pathway of (C_6_H_5_)PSSe–Cd–S intermediate. Data in the path are the relative Gibbs free energies (in kcal/mol and bond distances in angstroms) obtained at M06/6-31G(d) level

Decomposition of INT2/d along pathway 3 proceeds through a phenyl-dissociation transition state (TS4/d) in which the dissociation of the phenyl-radical is 3.85 Å away from the P atom. This process is associated with an activation barrier of +36.87 kcal/mol. The process is found to be exergonic, producing INT4/s at an energy level of 4.57 kcal/mol below the INT2/d. As outlined before [42], another plausible decomposition route occurs by the decomposition of phenyl group from the INT3/d. This pathway leads to the formation of INT5/s (shown in Fig. 3) passing through a doublet transition state, TS5/d accounts for the dissociation of the phenyl radical being 2.93 Å away from the associated P atom. INT5/s is produced at an energy level of 18.42 kcal/mol below the INT3/d. The phenyl-dissociation transition state, TS5/d, possesses an activation barrier of 32.83, ∼4 kcal/mol lower than pathway 3 discussed above.

It was reckoned that the (C_6_H_5_)PSSe–Cd–Se INT4/s intermediate produced in Scheme 1 may then decompose in two ways, either through the formation of CdSe or ternary CdSe_x_S_1−x_. The energetics of such reaction was investigated and it was found that the activation barrier and the reaction energy for the formation of CdSe through a singlet transition state is +73.97 and −29.86 kcal/mol, respectively. The formation of ternary CdSe_x_S_1−x_ has an activation barrier and a reaction energy of +71.43 and −26.83 kcal/mol, respectively. The activation barrier for the formation of the CdS by the dissociation of the Cd–S and Cd–Se bonds from (C_6_H_5_)PSSe–Cd–S INT5/s intermediate is +95.15 kcal/mol (Fig. 5). This is much higher than the barrier for the formation of the ternary CdSe_x_S_1−x_.

As shown in Figs. 2 and 3, the final decomposition pathways that were considered have a higher activation barrier. It is worth noting that the higher energy values of the transition states associated with the final pathways are consistent with the strained, four cantered nature of the calculated transition state structures. The lowest barrier (∼60 kcal/mol) on the potential energy surfaces is ternary CdSe_x_S_1−x_ dissociation pathway. A rate constant of 7.88 × 10^−7^ s^−1^, 1.86 × 10^8^ mol L^−1^ and 1.61 × 10^−4^ mol L^−1^ s^−1^ were estimated for this pathway (Table 2). In terms of energetic, the formation CdSe is the thermodynamically more stable product on the reaction PES (Fig. 2). The rate constant along this pathway is 1.86 × 10^8^ mol L^−1^ (Table 2). Though Opoku et al. [42] found the CdS-elimination pathway as the most favoured pathway and ternary CdSe_x_S_1−x_ elimination as the most disfavoured one in their calculation using Cd[(^*i*^Pr)_2_PSSe]_2_ analogue, the present study suggest the ternary CdSe_x_S_1−x_ formation pathway as the most favoured pathway followed by CdSe and CdS-elimination pathways among the several possible decomposition pathways discussed above for the gas-phase thermal decomposition of Cd[(C_6_H_5_)_2_PSSe]_2_ precursor.

**Table 2:** Calculated rate constants for gas phase decomposition of Cd[(C_6_H_5_)_2_PSSe]_2_ at 800 K

Reaction pathway	K_uni_ (s^−1^)	K_eq_ (mol L^−1^)	k_rec_ (mol L^−1^ s^−1^)
INT4/s → P1/s	8.68 × 10^−13^	1.86 × 10^8^	1.61 × 10^−4^
INT4/s → P2/s	1.10 × 10^−16^	5.12 × 10^3^	5.65 × 10^−13^
INT5/s → P3/s	9.84 × 10^−14^	1.12 × 10^6^	1.10 × 10^−7^
INT5/s → P4/s	7.88 × 10^−7^	1.13 × 10^6^	8.95 × 10^−1^
INT6/d → P5/s	4.23 × 10^−3^	7.64 × 10^6^	3.23 × 10^4^
INT6/d → P6/s	3.17 × 10^−1^	3.26 × 10^1^	1.03 × 10^1^
INT6/d → P7/s	6.20 × 10^−3^	7.64 × 10^6^	4.74 × 10^4^
INT7/s → P8/s	1.30 × 10^−3^	5.90 × 10^2^	7.69 × 10^−1^
INT7/s → P9/s	1.53 × 10^−3^	1.52 × 10^−2^	2.32 × 10^−5^
INT7/s → P10/s	1.47 × 10^−3^	7.92 × 10^−11^	1.16 × 10^−13^

**Fig. 4:**
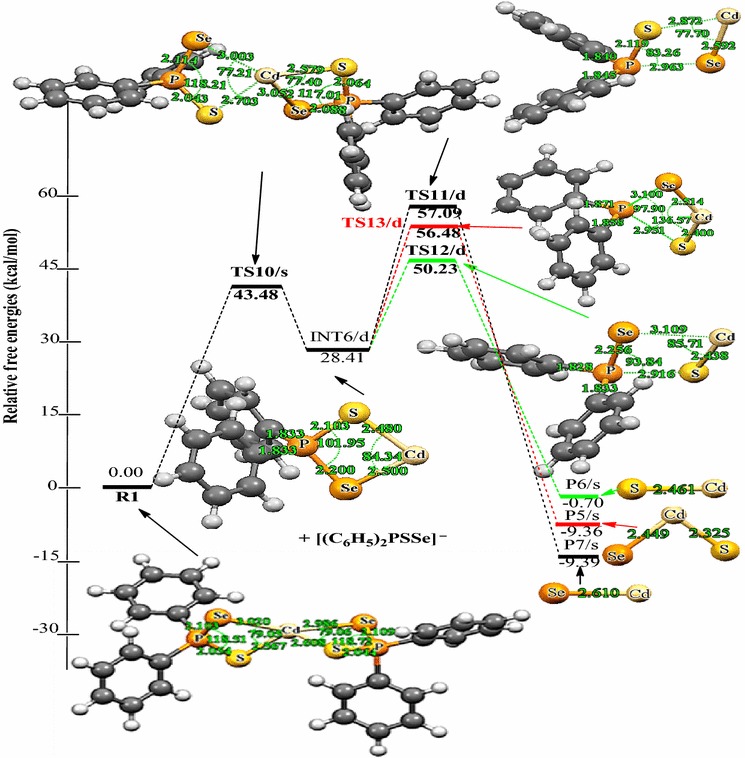
Energy profile of the decomposition pathway of (C_6_H_5_)_2_P(Se)S–Cd intermediate. Data in the path are the relative Gibbs free energies (in kcal/mol and bond distances in angstroms) obtained at M06/6-31G(d) level

As outlined before, another plausible decomposition route originating from R1 is Cd–Se and Cd–S elimination (Scheme 3). The fully optimized geometries of all the reactants, intermediates, transition states (TS), and products involved in the Cd[(C_6_H_5_)_2_PSSe]_2_ decomposition are shown in Fig. 4. Decomposition of R1 proceeds through the dissociation of Cd–Se and Cd–S bonds on one side of the ligand via a singlet transition state to form a (C_6_H_5_)_2_PSSe–Cd intermediate on the doublet PES, which is like the loss of a phenyl radical in Scheme 1. This process is associated with an activation barrier and a reaction energy of 43.48 and 28.41 kcal/mol above the initial reactant, R. The (C_6_H_5_)_2_PSSe–Cd intermediate, INT6/d, formed can enter into three successive reactions.

As shown in Fig. 4, further decomposition of INT6/d may lead to the formation of CdSe (shown in Scheme 3) through Cd–S and P–Se elimination. This passes through the transition state TS11/d and requires a barrier height of 28.68 kcal/mol above the INT6/d; the corresponding reaction energy is 37.80 below the reactant. The Cd–S bond elongates from 2.48 Å in the complex to 2.87 Å in the transition state, and the P–Se bond also elongates from 2.20 Å in the complex to 2.96 Å in the transition state.

Another subsequent elimination may follow from INT6/d and give rise to the formation of CdS with the elimination of Cd–Se and P–S bonds. The Cd–Se and P–S bond distances elongate from 2.50 and 2.10 Å in the complex to 3.11 and 2.92 Å in the transition state. This process requires a barrier height of 21.82 kcal/mol at TS12/d and free energy of −29.11 kcal/mol (Fig. 4). Therefore, the results suggest that the dissociation of CdS is kinetically preferred over the dissociation of CdSe.

A subsequent decomposition via INT6/d, leads to the formation of a ternary CdSe_x_S_1−x_. This process needs to go over a barrier of 28.07 kcal/mol (relative to INT6/d) via a doublet transition state TS13/d. The reaction is calculated to be exergonic by 37.77 kcal/mol (relative to INT6/d). The P–Se and P–S bonds elongate from 2.20 and 2.10 Å in the complex to 3.10 and 2.95 Å in the transition state.

Among the three possible heterolytic dissociations pathway, the CdSe dissociation pathway is slightly the most stable species on the reaction PES, with a free energy of about 0.03 kcal/mol lower than the CdS. The results suggest that, the heterolytic pathway of CdSe through the [(C_6_H_5_)_2_PSSe]^−^ anion is highly competitive with the CdS pathway. Moreover, in terms of kinetic, the CdS dissociation is the most favourable pathway than the CdSe and ternary CdSe_x_S_1−x_ pathways and a rate constant of 3.17 × 10^−1^ s^−1^ was estimated (Table 2).

**Fig. 5:**
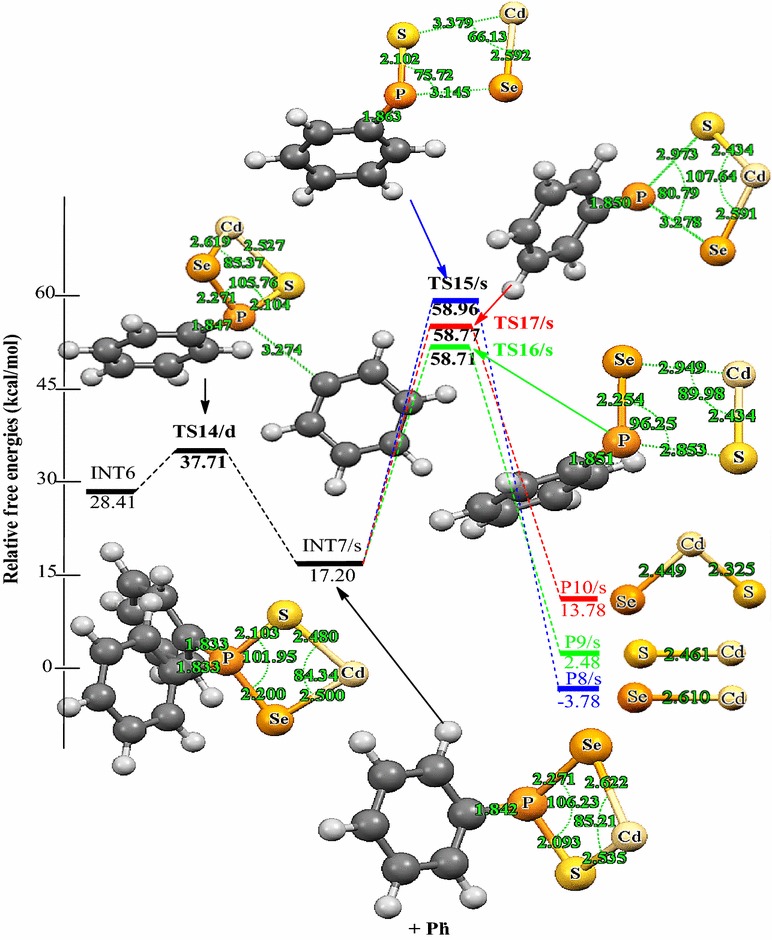
Energy profile of the decomposition pathway of (C_6_H_5_)P(Se)S–Cd intermediate. Data in the path are the relative Gibbs free energies (in kcal/mol and bond distances in angstroms) obtained at M06/6-31G(d) level

The (C_6_H_5_)_2_PSSe–Cd intermediate, INT6/d thus formed, is widely believed to be an important precursor for the growth of the cadmium chalcogenides. Understanding the decomposition of INT6/d is therefore crucial in order to gain important insight into the complex gas-phase mechanism leading to the identification of intermediates on the singlet PES (Scheme 4). The relative free and activation energy of the main stationary points involved in Scheme 4 are shown in Fig. 5. The dissociation of phenyl radical through a doublet transition state TS14/d to form a (C_6_H_5_)P(Se)S–Cd intermediate, INT7/s on a singlet PES has an activation barrier of +9.30 kcal/mol and exergonic by 11.21 kcal/mol.

As shown in Scheme 4, decomposition of INT7/s may proceed via three pathways, all of which lead to the removal of carbon contamination through the elimination of carbon containing fragments. The decomposition pathway, going through the TS15/s transition state with a barrier height of 41.76 kcal/mol, is a CdSe elimination process which involves the dissociation of Cd–S and P–Se bonds from INT7/s. The CdSe product is located at 20.98 kcal/mol below the reactant.

Decomposition of INT7/s may also proceed through a singlet transition state, TS16/s, having an activation barrier of 41.51 kcal/mol and exergonicity of 14.72 kcal/mol. This leads to the formation of CdS resulting from the elimination of Cd–Se and P–S bonds.

In an alternate dissociation route involving the dissociation of P–S and P–Se bonds, INT7/s gives rise to the formation of a ternary CdSe_x_S_1−x_. This process is associated with an activation barrier of 41.57 kcal/mol and passes through a singlet transition state TS17/s. The resulting product being 3.42 kcal/mol below INT7/s is ∼18 and ∼11 kcal/mol less stable than the CdSe and CdS dissociation pathway, respectively.

However, CdSe is comparable, located only at 0.25 and 0.19 kcal/mol higher than CdS and ternary CdSe_x_S_1−x_. Therefore one of the three pathways is not overwhelming to the other but instead competing even if CdS dissociation is a little more favourable. The rate constants along CdS pathway were 1.53 × 10^−3^ s^−1^ and 2.32 × 10^−5^ mol L^−1^ s^−1^ (Table 2). Moreover, all the reactions were predicted to be exergonic, ranging from ~ 3–21 kcal/mol. However, the results further suggested that the formation of CdSe is the most stable species on the reaction PES.

In order to provide a direct comparison of activation energy data for a phenylphosphinato complex and its isopropyl analogue, the Cd[(C_6_H_5_)_2_PSSe]_2_ complex was prepared as a model for Cd[(^*i*^Pr)_2_PSSe]_2_ complex. Precedent for the modelling of phenyl complex is provided by the virtually identical decomposition patterns for the isopropyl complex [42]. DFT results for phenyl group could then be compared to our previously reported data for the isopropyl complex [42]. The activation barrier and reaction energy of the two precursors are presented in Table 3.

**Table 3:** Calculated activation barriers and reaction energy of the last step of the various reactions of the Cd[(C_6_H_5_)PSSe]_2_ and Cd[(^i^Pr)_2_PSSe]_2_ complexes

Reaction pathway	Activation barrier		Reaction energy	
INT4/s → P1/s	+73.97	+33.65^b^	−29.86	−30.92^b^
INT4/s → P2/s	+71.43	+41.35^b^	−26.83	−44.82^b^
INT5/s → P3/s	+95.15	+29.87^b^	−16.72	−26.21^b^
INT5/s → P4/s	+59.08	+59.65^b^	−19.92	−48.43^b^
INT6/d → P5/s	+26.68	+27.66^b^	−37.80	−21.56^b^
INT6/d → P6/s	+21.82	+29.90^b^	−29.11	−14.85^b^
INT6/d → P7/s	+28.07	+46.52^b^	−37.77	−34.27^b^
INT7/s → P8/s	+41.76	+12.83^b^	−20.98	−22.29^b^
INT7/s → P9/s	+41.51	+34.94^b^	−14.72	−13.97^b^
INT7/s → P10/s	+41.57	+20.94^b^	−3.42	−14.84^b^

^b^Data from Opoku et al. [38]

The kinetics and thermodynamics of organic and inorganic substituents, and radical reaction pathways may be affected by the size of structural features of either the substrate or the dissociation species. Since any homogeneous decomposition of electron transfer reaction requires appropriate orbital overlap, features that diminish such overlap will reduce the corresponding rate constants. Increasing substitution across the phosphinato complex, increases the activation barrier of the phenyl group, which are significantly greater than the isopropyl analogue. This suggests that the steric congestion afforded by this bulky substituent imposes significant energy on the electron transfer processes. Thus increased alkyl substitution may increase the chemical reaction of the decomposition process and decrease the activation barrier. Therefore, the kinetic stabilities of the resulting ligands depend on the steric congestion about the central phosphorus; more congested compounds are resistant to decomposition, while those with more accessible phosphorus centres react rapidly.

Moreover, the activation barrier data of the phenyl and isopropyl group may also suggest that the *C*–*Ph* bond is more difficult to break than the *C*–^*i*^*Pr* bond. This is consistent with the homolytic bond strength of the *C*–^*i*^*Pr* moieties [42]. If *C*–^*i*^*Pr* bond cleavage were involved in the rate determining step, phenyl complex would be expected to require higher deposition temperatures relative to the isopropyl complex. The stronger *C*–*Ph* bond may also affect growth rate and composition of the deposited films. Additionally, these data suggest that replacing the phenyl moiety with a group that will cleave more readily could decrease the deposition temperature and improve the compositional characteristics of the cadmium chalcogenides films.

### Spin density analysis

The spin density distribution map of some intermediates and transition states complexes obtained on the doublet PES has been explored on the M06/6-31(d) level of theory.

**Fig. 6:**
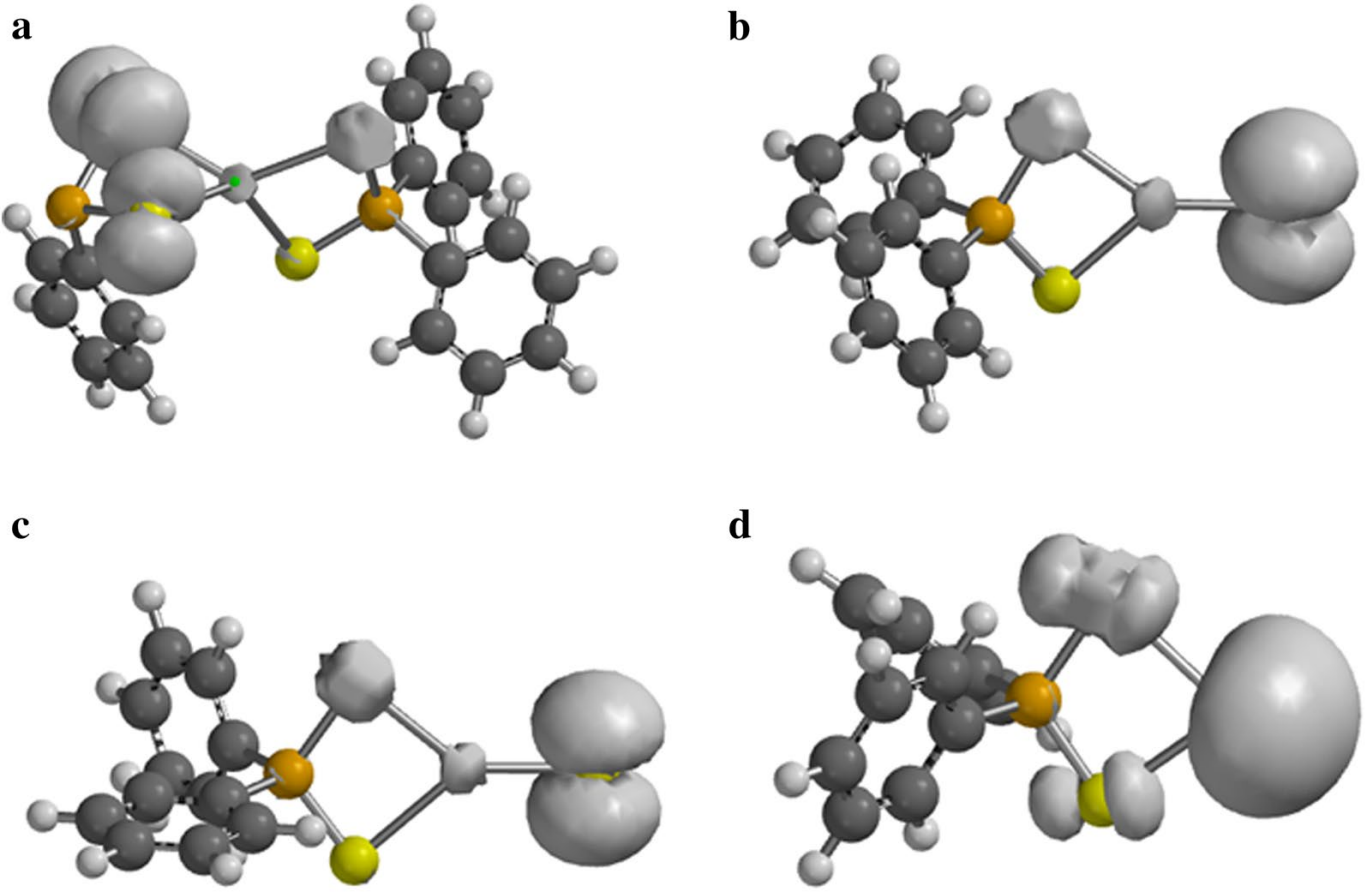
Spin-density distribution for **a** (C_6_H_5_)_2_PSSe–Cd–SeSP(C_6_H_5_), **b** (C_6_H_5_)_2_PSSe–Cd–Se, **c** (C_6_H_5_)_2_PSSe–Cd–S and **d** (C_6_H_5_)_2_PSSe–Cd complexes. Isosurfaces ± 0.003 a.u

In Fig. 6a–c, most of the spin densities are distributed on the ligand with less metal contribution. As shown in Fig. 6d, the spin density is entirely distributed on the cadmium atom that coordinates to the ligand.

**Fig. 7:**
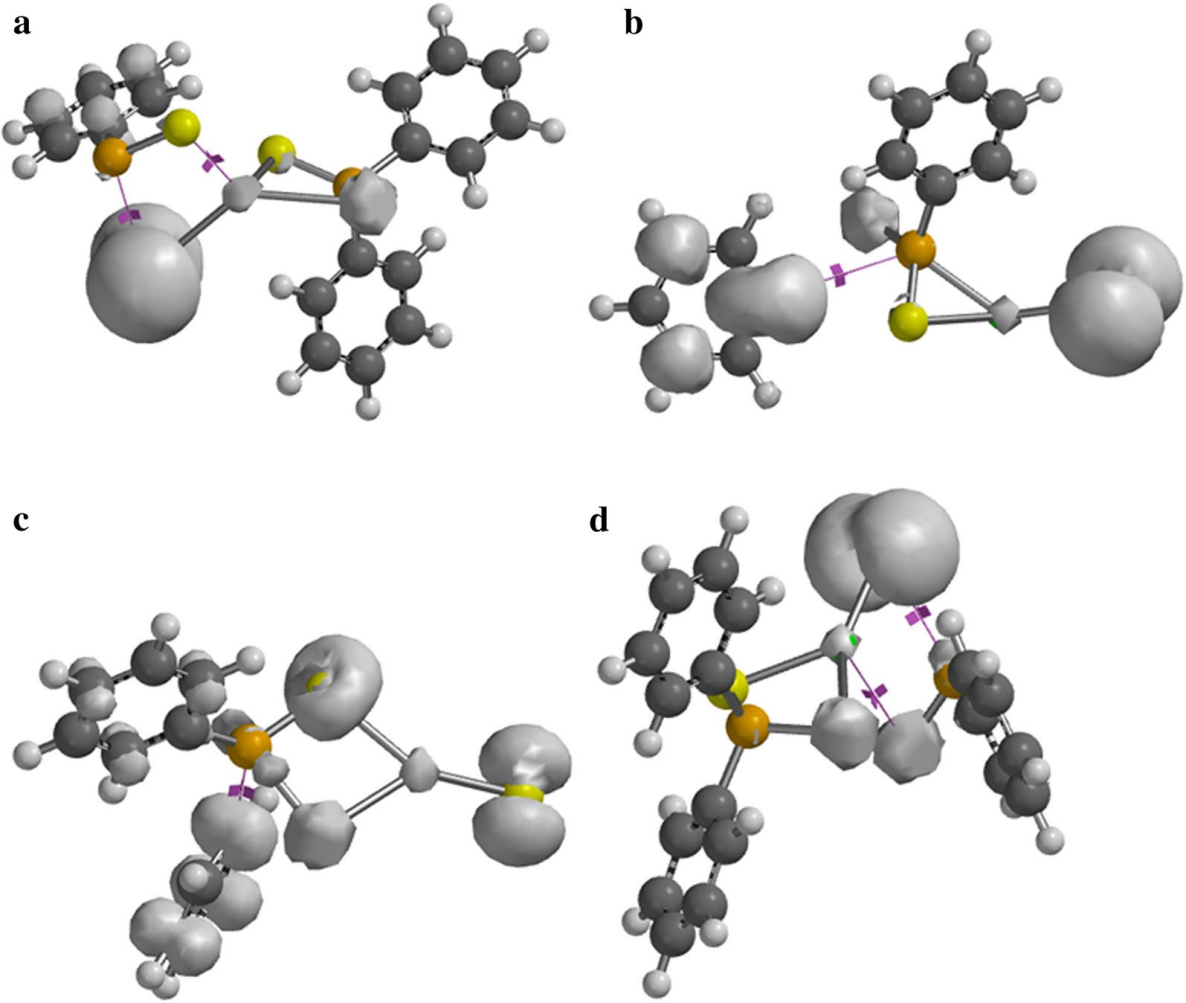
Spin-density distribution for **a** (C_6_H_5_)_2_PSSeCdSe.SP(C_6_H_5_), **b** C_6_H_5_.(C_6_H_5_)PSSeCdSe, **c** C_6_H_5_.(C_6_H_5_)PSSeCdS complexes and **d** C_6_H_5_)_2_PSSeCdS.SeP(C_6_H_5_), (**c**). Isosurfaces ± 0.003 a.u.

In Fig. 7a–c, additional spin density is symmetrically delocalised on the phenyl group with little or no spin on the phosphorus atom.

**Fig. 8:**
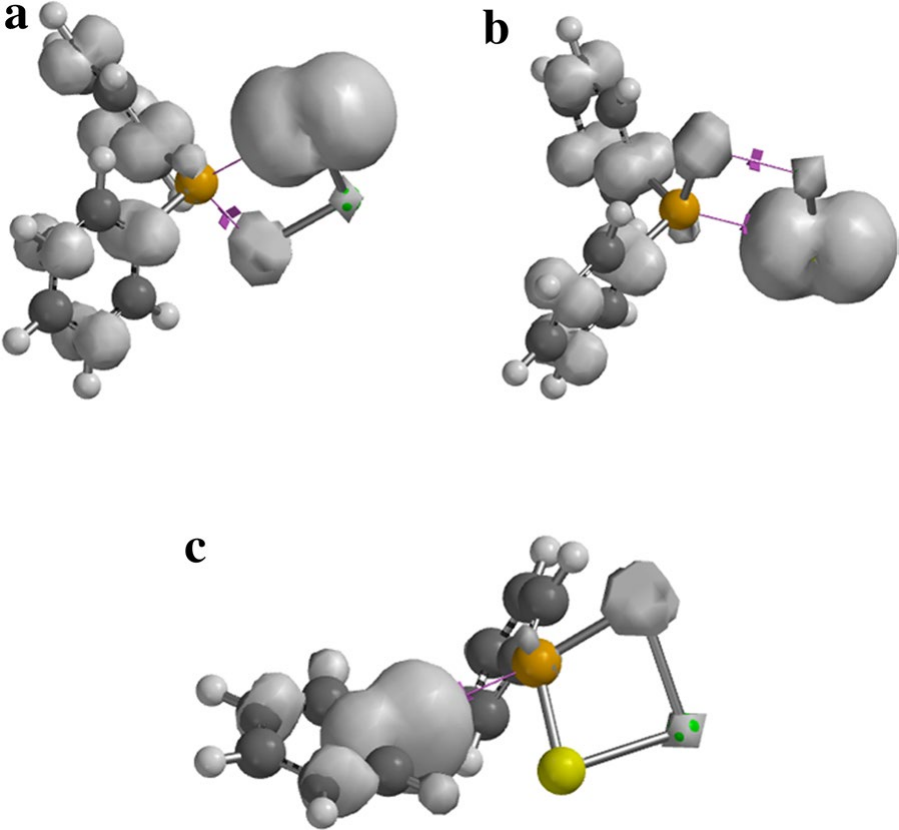
Spin-density distribution for **a** (C_6_H_5_)_2_P.CdSeS, **b** (C_6_H_5_)_2_PSe.CdS and **c** C_6_H_5_.(C_6_H_5_)PSSeCd complex. Isosurfaces ± 0.003 a.u.

In Fig. 8a–c, the spin density is exclusively localised on the selenium atom with less metal contribution. Additional spin density is symmetrically delocalised on the phenyl group that coordinate to the phosphorus atom.

### Orbital analysis

**Fig. 9:**
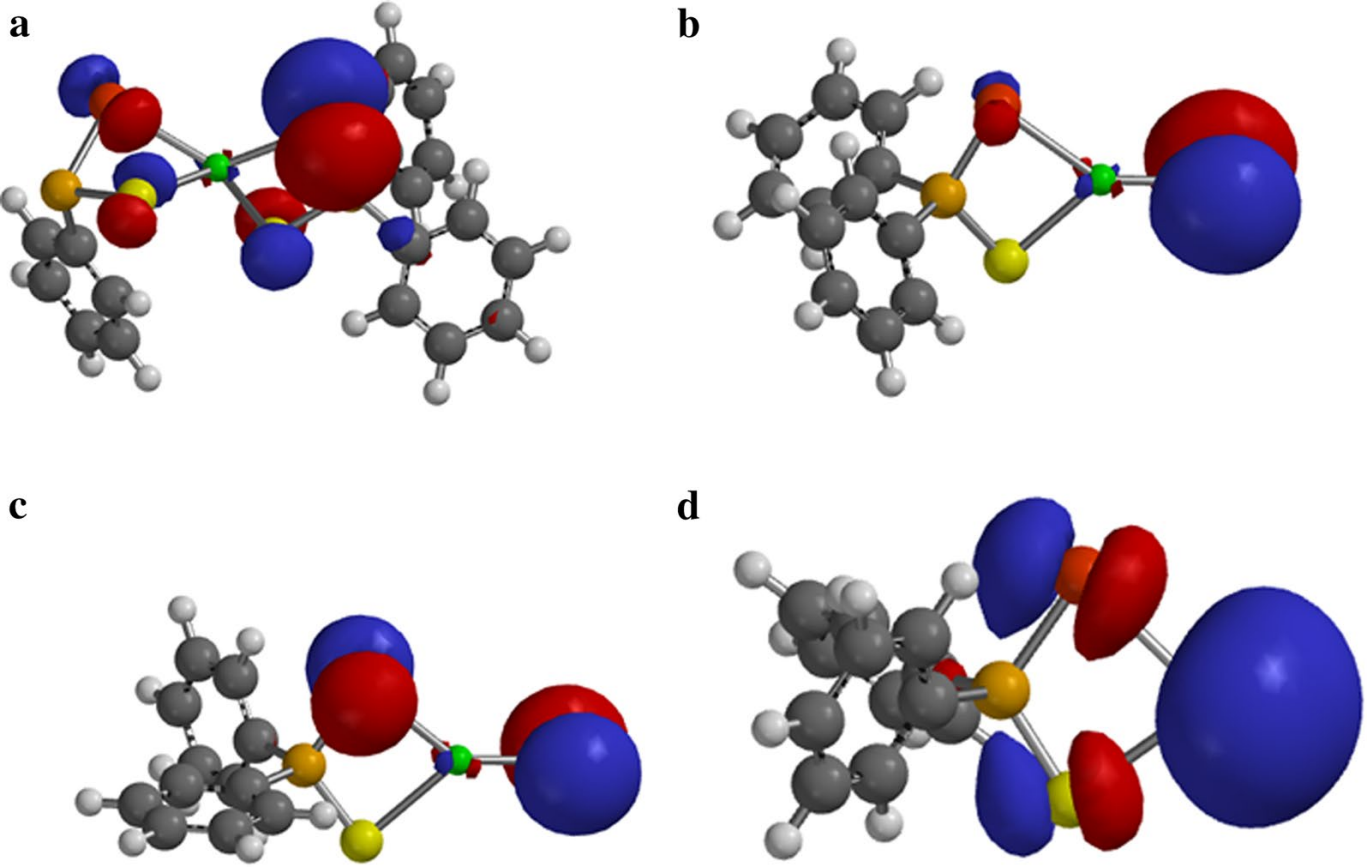
Singly occupied molecular orbitals for **a** (C_6_H_5_)_2_PSSe–Cd–SeSP(C_6_H_5_), **b** (C_6_H_5_)_2_PSSe–Cd–Se, **c** (C_6_H_5_)_2_PSSe–Cd–S and **d** (C_6_H_5_)_2_PSSe–Cd complexes. Isosurfaces ± 0.032 a.u.

The single occupied molecular orbital (SOMO) analysis of some intermediates and transition states complexes has also been explored at the same level of theory. In Fig. 9a–c, the electron density distribution on the cadmium atom resembles that of d-xy orbital; a significant contribution from the ligand was also observed. The SOMO of (C_6_H_5_)_2_PSSe–Cd^+^ complex shows a strong localisation of electron density on the cadmium atom as compare to the ligand.

## Conclusion

The Cd[(C_6_H_5_)_2_PSSe]_2_ complex was tested to determine its suitability as a single-source precursor for cadmium chalcogenides thin films. The decomposition of Cd[(C_6_H_5_)_2_PSSe]_2_ as a single source precursor, is investigated using density functional theory at the M06/LACVP* level. Kinetically, the dominant pathways for the gas-phase decomposition of Cd[(C_6_H_5_)_2_PSSe]_2_ were found to be CdS elimination pathways on both the singlet and the doublet PESs. However, on the basis of the dissociation energy of the reactions and with the detailed identification of the reaction intermediates, it is clearly shown that CdSe elimination pathways are the dominant pathways on both the singlet and the doublet PESs. Comparison of energetics of the phenyl group to the isopropyl analogue, allows evaluation of the effect of the phosphinato bond dissociation energy on final decomposition products. The isopropyl precursor is superior to phenyl for barrier deposition due the tendency of the stronger phosphinato bond of phenyl to result in dissociation of the *C–Ph* fragments. The exploration of chemical kinetics and the construction of global potential energy surfaces for the decomposition of SSPs are believed to provide a comprehensive fundamental molecular level understanding of the reaction mechanism involved in the chemical vapour deposition.
